# Erythrocyte glutathione transferase in kidney transplantation: a probe for kidney detoxification efficiency

**DOI:** 10.1038/s41419-018-0289-3

**Published:** 2018-02-19

**Authors:** Alessio Bocedi, Annalisa Noce, Valentina Rovella, Giulia Marrone, Giada Cattani, Massimo Iappelli, Paolo De Paolis, Giuseppe Iaria, Daniele Sforza, Mariacarla Gallù, Giuseppe Tisone, Nicola Di Daniele, Giorgio Ricci

**Affiliations:** 1 Department of Chemical Sciences and Technologies, University of Rome, Tor Vergata, Via della Ricerca Scientifica, 00133 Rome, Italy; 2 Department of Systems Medicine, Hypertension and Nephrology Unit, University of Rome, Tor Vergata, Viale Oxford 81, 00133 Rome, Italy; 3 Nephrology and Transplant Unit, A.O.S. Camillo-Forlanini, Circonvallazione Gianicolense 87, 00152 Rome, Italy; 4 Liver and kidney Transplant Centre, University of Rome, Tor Vergata, Viale Oxford 81, 00133 Rome, Italy

## Abstract

Erythrocyte glutathione transferase (e-GST) is overexpressed in case of increased blood toxicity and its level correlates with the kidney disease progression. Thus, it represents a probe of kidney efficiency against circulating toxins. We measured the activity of e-GST in patients with transplant kidney from living and cadaver donors, correlated its level to biochemical parameters of kidney function, and measured the level of oxidized albumin as a probe of oxidative stress using a new simple procedure. Interestingly, the activity of e-GST in transplant patients from cadaver donors (*N* = 153) is very high (11.7 U/g_Hb_) compared to healthy subjects (*N* = 80) ( 5.6 U/g_Hb_). Lower values were observed in transplant patients with kidney from living donors (*N* = 16) (9.8 U/g_Hb_). Except for steroids, no correlation has been found with the immunosuppressive therapies and routine clinical and laboratory parameters. Also serum oxidized albumin, which reveals oxidative stress, is significantly higher in transplant patients from cadaver donors (53%) compared to that from living donors (36%). Overall, these data indicate that most of transplant kidneys from cadavers lost part of the detoxifying power against circulating toxins and suffer a relevant oxidative stress compared to those coming from living donors. A case report suggests that e-GST could represent a very early marker of incipient graft rejection. In conclusion, e-GST may be used to check the decline or maintenance of the kidney detoxification competence during post-transplantation course.

## Introduction

Kidney transplantation (KT) represents the treatment of choice in end-stage renal disease (ESRD) patients^1^. KT improves the quality of life and decreases the cardiovascular mortality and morbidity, compared to dialysis^1–5^. For example, about 20 years ago a large study involving 220,000 patients receiving long-term dialysis or cadaveric transplant demonstrated larger benefits about mortality in transplant patients after 18 months from the graft^1^. Also, a more recent study showed that KT is the most safe method among renal replacement therapies in patients with polycystic kidney disease because it is associated with a low morbidity and a high patient survival rate^6^. These results have also been highlighted even in patients older than 70 years; in fact the transplantation in these patients offers survival advantages over dialysis treatment^7^. New advances in immunosuppressive therapy induced a decrease in cases of acute rejection and improved graft survival^8,9^. An accurate correlation between immunosuppressive dosage and the outcome of transplant kidney is required in order to provide adequate pharmacological treatment ^9^. The imprecision and inaccuracy in the predictiveness of diagnostic biochemical markers currently in use, makes pharmacological treatment more difficult^10^. To date, no biomarkers able to assess the ability of the transplanted kidney in removing many different toxic compounds are available. The present study focuses on a first application of erythrocyte glutathione transferase (e-GST) as a useful tool to check the competence of transplanted kidneys in this specific role. e-GST (synonym of human glutathione transferase P1-1 (GSTP1-1)) is an homo-dimeric intracellular enzyme of about 46 kDa that represents the most abundant form of intra-erythrocyte transferase (i.e., 95% of an entire GST pool)^11^. More in general, glutathione transferases (GSTs) are a superfamily of iso-enzymes able to promote the conjugation of glutathione with many toxic compounds of different chemical nature and shapes, and thus favoring their excretion^12^. Human cytosolic GSTs have been grouped into seven gene independent classes (i.e., Alpha, Mu, Pi, Theta, Omega, Sigma, and Zeta). In addition, GSTs may act like ligandins by sequestering many small and/or large toxic compounds and peptides. Furthermore, GSTP1-1 appears to control signaling pathways and transcriptional responses of cells through protein–protein interaction mechanisms^13^. GSTs are also able to bind the dinitrosyl-diglutathionyl–iron complex, a natural toxic derivative of nitric oxide, which becomes harmless when bound to GST^14^. Besides classical transcriptional activators (e.g., xenobiotics and anticancer drugs), *GSTP* gene is modulated by the levels of endogenous activators. These include toxins produced by the functional defect of depurative organs such as liver and kidney^15^. In this context, e-GST, compartmentalized in the erythrocytes and not dialyzable, seems to be an interesting long-term biomarker either to assess kidney function and also the adequacy of dialytic techniques^16^. e-GST assay represents a very simple, inexpensive, and fast laboratory tool in clinical nephrology also developed for high-throughput routine protocols^16,17^. e-GST may be considered a sort of ideal long-term biomarker that should provide a measure of circulating toxins in temporal spans not limited to a single day or dialysis session but extended up to one/two months^16^. The e-GST assay, specific and accurate for this enzyme^18^, has been applied in several studies concerning validation and use of e-GST as biomarker in hemodialysis patients^17^ and in the assessment of dose and adequacy of dialysis therapy^19^. Moreover, e-GST was found to be overexpressed in chronic kidney disease (CKD) patients under conservative therapy^17,20^ and in type-2 diabetes mellitus patients (T2DM) with CKD^21,22^. Finally, environmental^23^ and endogenous factors like autoimmune disease^24^ also affected e-GST levels in healthy subjects and in non-uremic patients. In this study, we use e-GST like a probe able to assess functionality of transplant kidneys coming from living and cadavers donors in their specific action to scavenge endo and exogenous toxins. In addition, we also adopted a very simple and inexpensive procedure to evaluate the amount of oxidized albumin (HSAox) in serum of transplant patients. This protein shows 17 disulfide bridges and only one reduced cysteine (Cys34). This residue can react with serum disulfides to give glutathionylated or cystationylated protein. A few amount of this residue may also give higher oxidized forms such as sulphinic and sulfonic derivatives. This oxidized protein represents a reliable short-term biomarker of oxidative stress related to pathological conditions^25–27^. Interestingly, HSA is considered the major plasma protein target of oxidative stress in uremia^28^. In fact, relevant increase of this oxidized protein has been found in hemodialysis patients^25,29^, in CKD patients under conservative therapy^30^ but never evaluated in kidney transplant patients. Our data indicate that both e-GST and HSAox are higher than those found in healthy subjects, in particular in kidney transplant patients from cadavers. The levels of these two biomarkers suggest that all transplant kidney both from living and cadaver donors loose part of their detoxifying ability against circulating toxins.

## Results

### Patients enrollment and laboratory parameters assessment

The number of patients enrolled in the present study were: 153 kidney transplants from cadaver donors and 16 kidney transplants from living donors. The epidemiological features were summarized in Table 1. The two groups of patients (transplants from cadaver donors and transplants from living donors) were homogeneous for age and BMI. Several laboratory parameters were analyzed in two groups but we found statistical significance only for creatinine, estimated glomerular filtration rate (eGFR), and albumin (Table 2).

**Table 1:** Epidemiological features of transplant patients

	Transplant patients from cadaver	Transplant patients from living donors
Number (*N*)	153	16
Male/female	107/46	11/5
Age (years)	55 ± 12^a^	51 ± 22^a^
BMI	25 ± 4^a^	24 ± 3^a^
Time transplant (months)	70 ± 10^b^	48 ± 10^b^
Delayed graft function (%)	20.2	0
Diabetes mellitus (%)	16	4
Arterial hypertension (%)	87	72
Primitive cause of ERSD:		
(a) Glomerulonephritis (%)	36	39
(b) Nephroangiosclerosis (%)	20	15
(c) ADPKD (%)	13	12
(d) Chronic pielonephritis (%)	5	4
(e) Other causes (%)	26	30

*BMI* body mass index, *ESRD* end-stage renal disease, *ADPKD* autosomal dominant polycystic kidney disease

^a^ Data are expressed as mean ± SD

^b^ Data are expressed as mean ± SEM

**Table 2:** Laboratory parameters of transplant patients

	Transplant patients from cadaver	Transplant patients from living donors	*P*-value
Creatinine (mg/dl)	1.7 ± 0.8	1.3 ± 0.3	0.0224
eGFR (ml/min)	50 ± 2	65 ± 4	0.0029
Albumin (g/dl)	4.0 ± 0.4	4.5 ± 0.3	0.0002

Data are expressed as mean ± SEM. *P*-value < 0.05 is considered significant

*eGFR* estimated glomerular filtration rate

### e-GST level in transplant patients

**Fig. 1:**
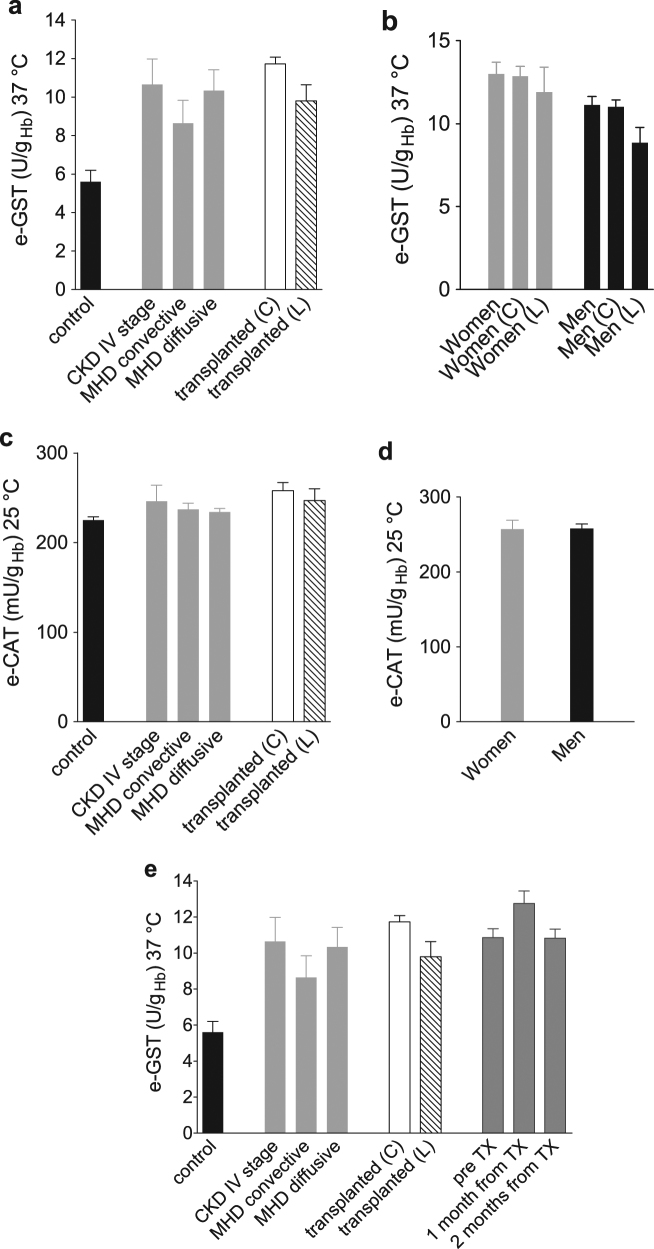
e-GST and e-CAT activity in all transplant patients. **a** Histogram of e-GST activity of transplant patients from cadaver (*N* = 153) (white bar) and living donors (*N* = 16) (streaked bar) compared to control group (black), and e-GST activity in chronic kidney disease stage IV patients^17^ and in patients under two different dialysis techniques^19^ (gray bars). Statistical significance among e-GST activities in control group and transplanted patients (control vs. transplanted from cadavers, *P* < 0.0001; control vs. transplanted from living, *P = *0.0008 and transplanted from cadavers vs. transplanted from living, ns *P* = 0.2120) **b** e-GST activity of all transplant patients divided by sex (women in gray and men in black) with a statistical significance of *P* = 0.013. The differences between sex are also reported for the type of donors: cadaver women (C) and men (C) and living women (L) and men (L). **c** Histogram of e-CAT activity of transplant patients from cadaver (*N* = 153) (white bar) and living donors (*N* = 16) (streaked bar) compared to control group (black), and e-CAT activity in chronic kidney disease stage IV patients^17^ and in patients under two different dialysis techniques^19^ (gray bars). No one of the observed differences showed statistical significance (for all groups *P* > 0.05). **d** e-CAT activity of all transplant patients divided by sex (women in gray and men in black). **e** Histogram of e-GST activity in eight patients before kidney transplantation (TX), 1 and 2 months after transplantation (gray bars) from deceased donors. Control group is the same for the experiments reported in panel **a** and panel **e**. Statistical significance among e-GST activities in control group and patients before transplantation (pre TX), one/two months after transplantation (1 month from TX and 2 months from TX) is not reported due to the scarce number (only eight) of patients. Values in the four panels are reported as mean ± SEM

e-GST level was determined in 153 kidney transplant patients from cadaver and 16 kidney transplant patients from living donors, at least 3 months from the transplantation. The results shown in the present study were compared with data of healthy control group (5.6 ± 0.6 U/g_Hb_), nephropathic patients under conservative therapy (stage IV according to KDIGO guidelines) (10.7 ± 1.3 U/g_Hb_), and hemodialysis patients under diffusive (10.3 ± 1.1 U/g_Hb_) and convective techniques (8.6 ± 1.2 U/g_Hb_)^21^ (Fig. 1a). The values of transplant patients from living donors (9.8 ± 0.8 U/g_Hb_) are close to those found in patients with maintenance hemodialysis with diffusive technique or in CKD patients (stage IV) (Fig. 1a). Moreover, values of transplant patients from deceased donors (11.7 ± 0.4 U/g_Hb_) are higher when compared to other kidney pathology recipients (Fig. 1a). The level of e-GST in transplant patients was higher in women (13.0 ± 0.7 U/g_Hb_) compared to men (11.1 ± 0.5 U/g_Hb_) (Fig. 1b), as also found in healthy subjects^15^. Unlike what observed for e-GST, erythrocyte catalase (e-CAT) levels of kidney transplant patients (from living and cadaver donors as well) were very similar in all other categories examined (e.g., healthy subjects, CKD patients under conservative therapy (stage IV), maintenance hemodialysis patients under convective and diffusive techniques) (Fig. 1c). Furthermore, no differences were underlined for e-CAT levels in women and men transplant patients (Fig. 1d). This last experimental evidence suggests a role of e-CAT as a constitutive control enzyme within the erythrocyte. A supplementary analysis of e-GST activity was performed on eight patients (six women/two men; mean age 56.4 ± 3.1 years) before KT, 1 and 2 months after transplantation (Fig. 1e). Notably, all eight patients were transplanted with kidneys from cadaveric donors. The average level of e-GST for these eight patients was 10.9 ± 0.5 U/g_Hb_ before transplantation, with an increase to 12.7 ± 0.7 U/g_Hb_ after 1 month and 10.8 ± 0.5 U/g_Hb_ (very similar to that before transplantation) after 2 months. Values before and after 2 months from transplantation were in the same range of e-GST levels compared to those measured for CKD IV stage (10.7 ± 1.3 U/g_Hb_), hemodialysis patients under diffusive technique (10.3 ± 1.1 U/g_Hb_). The value determined 1 month after transplantation was similar to that derived from transplant patients from deceased donors (11.7 ± 0.4 U/g_Hb_).

### e-GST level and estimated glomerular filtration rate

**Fig. 2:**
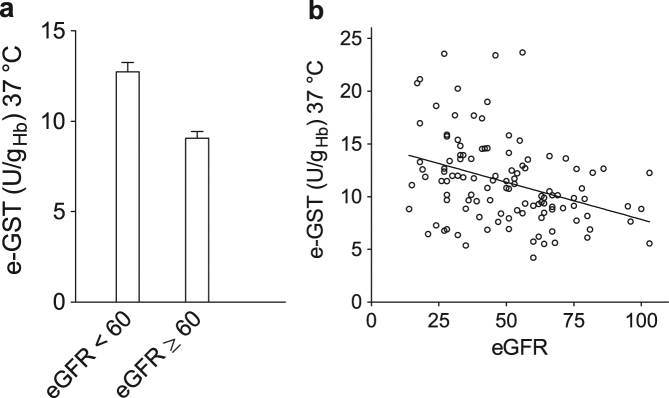
e-GST values in relation to eGFR parameter. **a** e-GST activity is reported in relation to the clinical parameter eGFR ≥ 60 ml/min and eGFR < 60 ml/min.e-GST activity is significantly increased in group with eGFR < 60 ml/min respect to the group with eGFR ≥ 60 ml/min (*P* < 0.0001). Values are reported as mean ± SEM. **b** Linear correlation between e-GST and eGFR for all the transplanted patients (*r*^2^ = 0.1330 and *P* < 0.0001)

The eGFR represents up to now the best index for kidney function^31^, even if this marker does not discriminate between toxic and non-toxic compounds. For assessment of eGFR, based on serum creatinine, it is recommended to use the CKD epidemiology collaboration (CKD-EPI 2009) equation^32,33^. The level of eGFR in healthy subjects is estimated to be ≥ 90 ml/min. According to Kidney Disease Quality Outcome Initiative (K-DOQI)^34^ and the Kidney Disease Improving Global Outcomes (K-DIGO)^35^ the diagnosis of CKD is formulated when eGFR is < 60 ml/min for >3 months with or without kidney disease or when kidney damage is defined by structural or functional abnormalities for >3 months with or without decrease of eGFR. The cutoff of 60 ml/min for eGFR has been chosen because this value is considered an early signal of renal disease and also the lower values are associated with high risk of cardiovascular mortality and morbidity^34,36^. For these considerations, we divided all transplant patients into two subgroups based on the eGFR values with a cutoff of 60 ml/min. A remarkable increase of e-GST level is showed in the patient recipient with eGFR < 60 ml/min (Fig. 2). The value of eGFR < 60 ml/min, index of kidney damage, was confirmed also by e-GST hyper-expression (12.7 ± 0.5 U/g_Hb_). The value of e-GST for patients with eGFR ≥ 60 ml/min is lower (9.1 ± 0.4 U/g_Hb_) than e-GST measured in patients with eGFR < 60 ml/min (Fig. 2a). Moreover, a moderate linear correlation is reported (Fig. 2b) but a univariate analysis of variance (ANOVA) with a covariate showed that the two likely homogeneous groups (living and cadaveric donors) displayed different values. In particular, e-GST values were statistically increased in cadaveric donors compared to living donors tested against eGFR (*P* = 0.03).

### Correlations among e-GST level and clinical parameters

Table 3 shows that no evident correlation is present among the e-GST level, some clinical parameters and specific markers used to assess kidney function (e.g., albuminuria, creatinine, and azotemia). The lack of correlation confirms that e-GST may be considered a novel biomarker, which reveals the specific competence of a functional kidney in eliminating a lot of different systemic toxins not detectable by all common kidney biomarkers.

**Table 3:** Correlation between e-GST and some clinical parameters

Parameters vs.	e-GST	
	*R* ^2^	*P*-value
Total-COL (mg/dl)	0.0350	0.57
HDL-COL (mg/dl)	0.0192	0.22
LDL-COL (mg/dl)	0.000002	0.99
Months from transplant	0.0008	0.79
Height (cm)	0.0396	0.05
BMI (Kg/m^2^)	0.0047	0.51
Age (years)	0.0026	0.62
Weight (Kg)	0.0029	0.61
Dialytic Age (months)	0.0058	0.72
Albumin (g/dl)	0.0074	0.44
Albuminuria (mg/24 h)	0.0141	0.60
Creatinine (mg/dl)	0.00003	0.96
RI	0.0165	0.25
Uric acid (mg/dl)	0.0028	0.62
AST (U/l)	0.0149	0.25
ALT (U/l)	0.0128	0.28
γ−GT (UI/l)	0.0007	0.81
Urea (mg/dl)	0.0165	0.21
Glycemia (mg/dl)	0.0302	0.09
Calcium (mg/dl)	0.0476	0.04
Phosphorus (mg/dl)	0.0449	0.06
Sodium (mEq/l)	0.0001	0.91
Potassium (mEq/l)	0.0028	0.61

*Total-COL* total-cholesterol, *HDL-COL* high-density lipoprotein cholesterol, *LDL-COL* low-density lipoprotein cholesterol, *BMI* body mass index, *RI* resistive index, *AST* aspartate transaminase, *ALT* alanine transaminase, *γ−GT* gamma-glutamyltransferase

### Correlations among e-GST level and immunosuppressive drugs treatments

**Fig. 3:**
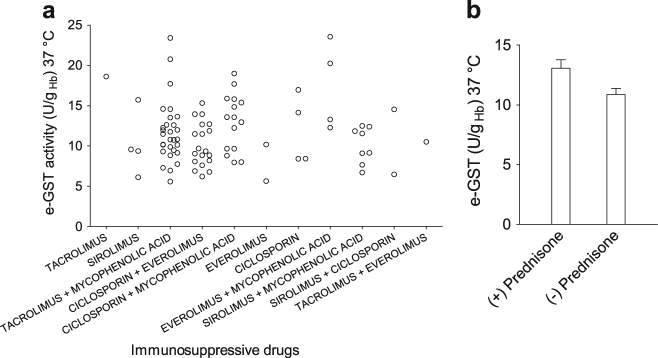
Correlation of e-GST activity with different immunosuppressive therapies. **a** e-GST activity estimated in 89 kidney transplant patients in relation with 11 different categories of “immunosuppressive drug cocktails” (mycophenolic acid represents both enteric-coated mycophenolate sodium and mycophenolate mofetil). **b** Histogram of e-GST values in relation to the corticosteroid (prednisone) administered during immunosuppressive therapies. Statistical significance (*P* = 0.0254) between e-GST activities in the group administered with prednisone (+) and without prednisone (−). Values are reported as mean ± SEM

Adequate immunosuppressive therapies are usually administered during post-transplant phase to avoid acute rejection and early graft loss. This therapy is usually constituted by the association of two or three drugs with different mechanism of action. Furthermore, each class of drugs is characterized by a different profile of toxicity. The choice of immunosuppressive treatment is based on both the recipient and donor clinical features. In this context, the possible correlations among immunosuppressive therapies and e-GST levels were studied. No evident correlations were found for e-GST levels and all immunosuppressive “drug combination” dispensed to the patients (Fig. 3a), although e-GST level was found slightly higher in patients treated with low doses of steroid (prednisone) compared to patients withdraw from steroid (respectively 13.1 ± 0.7 U/g_Hb_ vs. 10.9 ± 0.5 U/g_Hb_, *P* = 0.0254) (Fig. 3b). It has been shown that steroids play an important role in the development of insulin resistance. The latter is associated with altered metabolism in skeletal muscle, liver, and adipose tissue. Moreover steroids induced changes in cardiac metabolism, which translate into “lipotoxicity”^37,38^. Therefore, the slight e-GST hyper-expression could be not caused by steroid (prednisone) itself, but by the increase of hematic toxicity probably derived from the glyco-metabolic dysfunctions observed in patients in therapy with steroids. However, the patients withdrawn from steroid have an optimal kidney function and have a low risk of rejection.

### Case report

**Fig. 4:**
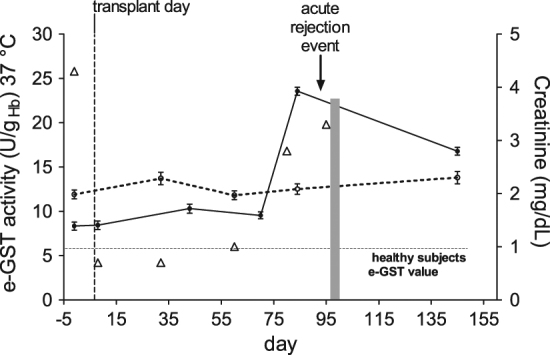
Observational study of e-GST level during a clinical history of a single patient. e-GST values (continuous line) are displayed during the clinical history on a single woman patient for 145 days. The acute rejection event occurred around day 95 (indicate by an arrow) was determined by histological examination obtained from biopsy. Mean value of e-GST level from seven patients examined during the same period (dashed line). Creatinine values (open triangle). Days of bolus of steroid administrated to the patient are reported in a light gray area. The e-GST level of healthy subject (5.6 U/g_Hb_) taken as a reference value is reported by an horizontal dotted line

Interestingly, an evident increase of e-GST level has been observed close to an acute rejection phase. During the present study, the value of e-GST was constantly monitored for around 5 months in eight transplant patients from cadavers. While slight increased values with time have been observed for seven samples (Fig. 4) (mean increase of e-GST = 16% in 5 months from transplantation), an e-GST increase of 180% was observed at day 84 for a woman of 69 years, who underwent acute rejection at day 95 (Fig. 4). The rejection was confirmed by histological examination, obtained from biopsy of the transplant organ, in collaboration with Pathology Anatomy Institute at Policlinico of University of Rome, Tor Vergata (data not shown). No other patients underwent acute rejection. After treatment of the woman with steroids bolus and recovery of renal function, e-GST decreased (Fig. 4). The evident increase of e-GST activity 11 days before rejection may be of great relevance as it proposes e-GST as a very early marker for graft rejection. Furthermore, an appreciable increase of creatinine was revealed during the rejection phase paralleling the e-GST increase (Fig. 4). Endovenous steroids bolus (prednisone 1000 mg) were administered from the 96th to 98th days after transplantation. Subsequently, the steroid therapy was administered orally for 45 days (0.5 mg/Kg body weight) with a following “tapering”.

### Oxidized serum albumin

**Fig. 5:**
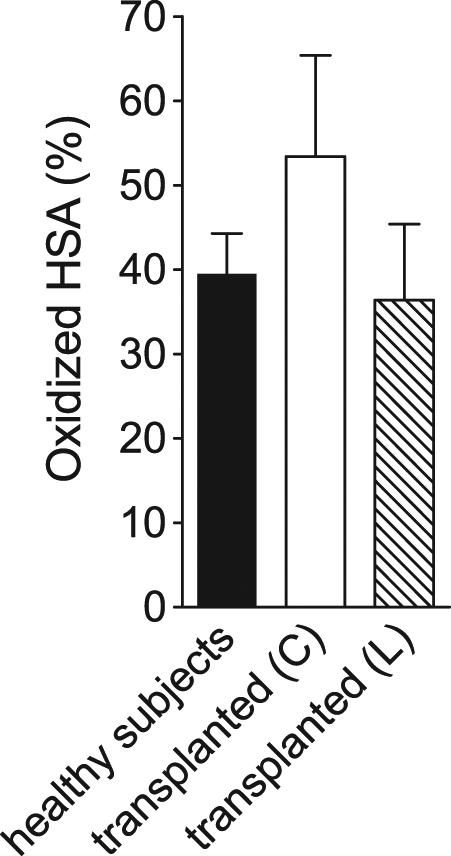
Oxidized serum albumin in kidney transplant patients compared to healthy subjects. Values of HSAox were determined for transplant patients from cadaver (*N* = 51) (white bar) and living donors (*N* = 16) (streaked bar) and compared to healthy subjects (*N* = 80) (black bar). Values are reported as mean ± SD. Statistical significance: control group vs. transplanted from cadavers, *P* = 0.0253; control group vs. transplanted from living, *P* = 0.0013; transplanted from cadavers vs. transplanted from living, *P* = 0.0093

The oxidized albumin, used as a short-term biomarker of oxidative stress, suggested the occurrence of relevant oxidative events in the transplant patients, particularly in those receiving kidneys from cadaveric donors (53.4 ± 12.0%) (Fig. 5). The amount of the oxidized protein in transplant patients with kidney coming from living donors (36.4 ± 9.0%) is like to that found in normal subjects (39.5 ± 4.8%). Our spectrophotometric procedure requires only a few minutes for analysis and allows the quantification of the entire pool of oxidized albumin forms without distinguishing between the three oxidized levels of Cys34 (i.e., mixed disulfide with serum thiols, sulphinic and sulfonic derivatives). The distinct levels of these forms, only measurable through expensive and time consuming methodologies, are not essential for the diagnosis of oxidative stress.

## Discussion

e-GST represents an efficient probe of long-term exposition to endogenous or exogenous toxins. Elevated levels of e-GST have been found in nephropathic patients as well as in healthy subjects living in polluted areas where hematic toxins are very high^16^. On this basis, the present study uses e-GST as an innovative biomarker able to evaluate the specific function of transplanted kidney against a lot of different toxins. Classical biomarkers like albuminuria, serum creatinine, and serum cystatin C only assess the renal function without specific indication about the ability of the kidney to remove toxic compounds. Conversely, e-GST level monitors the specific competence of the kidney to sweep many dangerous toxins^16,17^. In our study, e-GST activity was measured in 169 transplanted patients after at least 3 months from transplant and surprisingly e-GST levels were similar or even higher than that found in hemodialyzed patients indicating that circulating toxins are always high in both groups. This unexpected findings may be explained assuming that transplanted kidneys underwent an ischemia-reperfusion injury, occurring during retrieval, losing part of their detoxifying competence, more evident in the kidney coming from cadavers. We have observed delayed graft function (DGF) in 20.2% of transplant patients from cadavers and 0% from living donors (Table 1). DGF is defined as the need of dialysis during the first week after renal transplantation and it represents one of the most common early complications after KT^39^. The risk factors of DGF are both related to the recipient (such as ischemia-reperfusion injury, immunological response, immunosuppression therapy) and the donors (such as age and duration of both cold and warm ischemia)^40^. During renal reperfusion-ischemia, oxidative stress and inflammatory process have been demonstrated. In particular, kidney tissue injury during ischemia/reperfusion comes as a result of membrane lipids peroxidation, oxidative damage of proteins and DNA, and it results in apoptosis and necrosis^41^. Previous studies have been developed considering the cold ischemia time like a predictor of cardiovascular, nephro-urologic, and endocrine complications^42^. The observed increase of e-GST activity is an indication of paramount relevance as most of these kidneys display normal morphological structure and thus e-GST may represents an innovative and very sensitive biomarker of kidney efficiency. Overall, all these results may suggest that the better life expectancy found in transplant patients compared to dialyzed patients cannot be due to a lower level of circulating toxins or oxidizing compounds but, more likely, to the absence of frequent cardiovascular stress due to chronic dialytic therapy. In a second experimental section, we monitored eight patients in maintenance dialysis therapy, before KT, 1 and 2 months after transplantation (Fig. 1e). e-GST level increased 1 month after transplantation and returned to a baseline level after 2 months. We first hypothesized that the increased enzyme activity in the first month may be related to the “induction therapy” that is usually set immediately after transplantation in order to prevent acute rejection prior to maintenance therapy. This treatment typically includes the use of high doses of steroids and more specific T-cell direct therapy (i.e., thymoglobulin or basiliximab)^43^. However, in kidney transplanted patients for at least 3 months, no correlation has been found with the different immunosuppressive therapies and the e-GST levels, except a slight tendency toward significance with prednisone therapy (Fig. 3b). Thus, most of these drugs do not induce any hyper-expression of e-GST, that must be caused only by endogenous toxins. The level of plasmatic oxidized albumin is very interesting in this patients population. This protein is considered a short-term biosensor of oxidative stress and relevant changes of its level in plasma have been found before and after a single dialytic session^44^. Very high levels have been found in patients with transplanted kidney from cadavers while almost normal level in patients with transplanted kidney from living donors. This is a strong indication that oxidative stress is present only in the first recipient. Oxidized albumin is produced by oxidative stress, while e-GST is a biomarker for the presence of many different toxins but most of them do not induce oxidative stress; thus, the two biomarkers are not correlated. This may explain why in patients with transplanted kidney from living donors, the oxidized albumin is similar to healthy subjects (Fig. 5) while e-GST is higher (Fig. 1). Finally, we underline the case report that will be object of future investigations. The strong increase of e-GST level (about 180%) just before the occurrence of acute kidney rejection (Fig. 4) observed in a specific patient, could indicate e-GST as a sensitive predictive biomarker of incipient kidney rejection. This finding may be of interest given the absence of reliable biomarkers for this event. The observed rising of creatinine in the same period is usually considered a non-specific marker of graft dysfunction^45^. Moreover, there are limited data about the relationship between acute rejection episodes with altered albuminuria^46,47^. This may also explain the lack of correlation between e-GST and albuminuria observed in these patients as well as in all other patients (Table 3). Actually, more than 20 new biomarkers (i.e., Granzyme, FOXP3, etc.) have been proposed to be predictive tools for acute rejection, but none has been confirmed by robust validation studies^9,10^. Furthermore, most of them requires special equipment, are expensive and time consuming. Conversely, e-GST activity is cheap and can be performed in a few minutes with a simple spectrophotometer. In future, frequent e-GST measurements may be used to verify the decline or maintenance of the kidney detoxification competence during post-transplantation course. For example, it is well known that transplanted kidney are always less efficient during the first post-transplant period and this is also confirmed by increased GST levels (Fig. 1e). e-GST measurements may be useful to optimize pharmacologic strategies proposed to minimize this problem.

## Materials and methods

### Design of the study and patients

The protocol of this study followed the declaration of Helsinki and all patients healthy subjects provided a written informed consent. The present study was approved by the Ethical Committee of Azienda Ospedaliera Universitaria Policlinico Tor Vergata. A total of 169 patients were enrolled. Inclusion criteria were: age >18 years and renal transplant patients for at least 3 months. Early morning blood samples were taken from each patient for biochemical screening test after 12 h overnight fasting. Blood samples were collected into tubes (Vacutainer, BD, Plymouth, UK) containing ethylenediaminetetraacetate (EDTA). Blood was obtained via venipuncture from the antecubital vein. All samples were placed on ice and plasma was separated by centrifugation at 1600 × *g* for 10 min at 4 °C. Samples were stored with the same modality for all groups. e-GST, e-CAT, and HSAox were analyzed on the same blood sample within 1 h from delivery. Exclusion criteria in all kidney transplanted patients were active cancer and pregnancy, hyperbilirubinemia (e.g., Gilbert’s syndrome), virus hepatitis B and C, serum alanine aminotransferase and/or aspartate aminotransferase twice the upper limit of normal values, morbid obesity, rheumatologic disorders (e.g., systemic lupus erythematosus). Healthy subjects for the determination of HSAox and e-GST were 80 and 40 volunteers, respectively, from the transfusion medicine section of the Policlinico, University of Rome, Tor Vergata.

### Clinical parameters

An automated hematology analyzer XE-2100 (Sysmex, Kobe, Japan) was used for the determination of Hemoglobin (Hb). All routine parameters were determined using Dimension VISTA 1500 (Siemens Healthcare Diagnostics, Milano, Italy). The lipid profile (total-cholesterol (TC), triglyceride, low-density lipoprotein cholesterol, and high-density lipoprotein cholesterol) was determined by standard enzymatic colorimetric techniques (Roche modular P800, Roche diagnostics, Indianapolis, IN, USA). All other parameters were analyzed according to standard procedures in the Clinical Chemical Laboratories of the Policlinico, University of Rome, Tor Vergata.

### Estimated glomerular filtration rate

The eGFR was calculated using the CKD epidemiology collaboration (CKD-EPI) formula^32^.

### Chemicals and reagents

Glutathione (GSH), 1-chloro-2,4-dinitrobenzene (CDNB), ethylenediaminetetraacetic acid, H_2_O_2_, cystamine, 5,5′-dithiobis(2-nitrobenzoic acid) (DTNB) (Ellman’s reagent) and all other reagents were purchased from Sigma–Aldrich (St. Louis, MO, USA).

### Erythrocyte glutathione transferase (e-GST) activity

e-GST activity was obtained using a spectrophotometric procedure at 340 nm (37 °C), using an Uvikon 941 Plus spectrophotometer (Kontron Instruments, Watford, Herts, UK). One volume (40 μl) of whole blood was diluted into 1 ml of bi-distilled water causing fast erythrocytes hemolysis. Then, 100 µl were diluted to a final volume of 1 ml containing 1 mM GSH, 1 mM CDNB in 0.1 M potassium phosphate buffer, pH 6.5 according to Habig and coworkers^48^. Results were reported as enzyme units (U) per gram of hemoglobin (Hb) (U/gHb): one unit is the amount of enzyme that catalyzes the conjugation of 1 micromole of GSH to CDNB in 1 min at 37 °C^17^.

### Erythrocyte catalase (e-CAT) activity

e-CAT activity was determined with a spectrophotometric assay at 240 nm (25 °C) (Kontron Uvikon 941 Plus). Briefly, 5 μl of hemolyzed blood was diluted in 1 ml of buffer potassium phosphate 0.05 M pH 7.0 with EDTA 0.1 mM, and 10 μl of H_2_O_2_ 1 M according to Beers and Sizer^49^. Results were expressed as enzyme units (U) per gram of Hb (U/gHb): one unit is the amount of enzyme that catalyzes the decomposition of 1 micromole of H_2_O_2_ in 1 min at 25 °C.

### Oxidized human serum albumin (HSAox)

The HSAox was determined by subtracting the value of reduced HSA from the total HSA estimated from the routine clinical assay. The level of reduced HSA cannot be evaluated by direct titration of its reduced Cys34 by DTNB, because this thiol reagent reacts very slowly with this cysteine. We therefore used a modified procedure^50^ based on the fast reaction of cystamine with Cys34. The released cysteamine is stoichiometric with Cys34 and easily determined with DTNB (ε_412 nm_ of TNB^-^ : 14.1 mM^-1^ cm^-1^). The assay was carried on a Kontron Uvikon 941 Plus spectrophotometer (Kontron Instruments) at 412 nm (25 °C). One volume of 50 μl of serum was diluted in 0.89 ml of potassium phosphate buffer 0.1 M pH 8.0 recording an autozero sample. Suddenly, 50 μl of Ellman’s reagent (50 μM final concentration) and 10 μl of cystamine (1 mM final concentration) were added to the solution and after 15 min of incubation the absorbance was registered.

### Statistical and graphical analysis

Data are reported as means ± standard error of the mean (SEM). All continuous variables were checked for normality using Kolmogorov–Smirnov test. Differences between baselines and final values were tested (paired *t*-test and Mann–Whitney). The minimal level of significance of the differences was fixed at *P* < 0.05. One-way ANOVA was employed to compare the data between different data set of transplant patients (from living and cadaver donors). Comparison among groups was performed with the univariate ANOVA with a covariate for continuous parametric variables. Analysis of correlation was done with Pearson coefficient. This analysis was performed using the Statistical Package for the Social Sciences Windows, version 15.0 (SPSS, Chicago, Illinois, USA). The graphic and results visualization were obtained by GraphPad Prism (La Jolla, CA, USA).
